# Postoperative Joint Replacement Complications in Swedish Patients With a Family History of Venous Thromboembolism

**DOI:** 10.1001/jamanetworkopen.2018.1924

**Published:** 2018-09-07

**Authors:** Bengt Zöller, Peter J. Svensson, Jan Sundquist, Kristina Sundquist, MirNabi Pirouzifard

**Affiliations:** 1 Center for Primary Health Care Research, Lund University/Region Skåne, Malmö, Sweden; 2 Department of Coagulation Disorders, Skåne University Hospital, Lund University, Malmö, Sweden

## Abstract

**Importance:**

The associations of a family history of venous thromboembolism (FH-VTE) with postoperative venous thromboembolism (VTE) and major bleeding after joint replacement surgical procedures are unknown.

**Objective:**

To determine the risk of VTE and major bleeding in patients after primary hip or knee replacement surgical procedures.

**Design, Setting, and Participants:**

Cohort study using nationwide population-based databases of Swedish patients without a history of VTE who underwent joint replacement surgical procedures. Patients who had primary hip or knee replacement surgical procedures between July 1, 2005, and August 31, 2012, were identified. Patients born after 1931 without previous VTE were identified in the Swedish Multi-Generation Register. Only individuals with at least 1 parent and 1 full sibling alive between 1964 and the date for the surgical procedure were included. The data analysis was performed from September 1, 2017, to June 15, 2018.

**Exposures:**

Family history of VTE in a parent and/or a full sibling before the date of the surgical procedure.

**Main Outcomes and Measures:**

Venous thromboembolism and major bleeding within 90 days of the surgical procedure.

**Results:**

Of 69 505 study participants, 37 989 (54.7%) were women, and the median (interquartile range) age at the date of discharge was 65 (59-70) years. A total of 803 of 69 505 (1.2%) patients experienced postoperative VTE and 1285 (1.8%) experienced major bleeding. The cumulative VTE risk for those with FH-VTE was 231 of 15 858 (1.5%) and for those without an FH-VTE was 572 of 53 647 (1.1%) (*P* < .001). The cumulative bleeding risk for those with FH-VTE was 261 of 15 858 (1.6%) and for those without an FH-VTE was 1024 of 53 647 (1.9%) (*P* = .03). There was an association of patients with an FH-VTE who had increased VTE risk (adjusted hazard ratio [HR], 1.36; 95% CI, 1.17-1.59) and reduced bleeding risk (adjusted HR, 0.84; 95% CI, 0.74-0.97). There was an interaction between time after discharge and FH-VTE regarding VTE and major bleeding. An FH-VTE was not associated with VTE after discharge during the first week (HR, 1.13; 95% CI, 0.86-1.49). After 7 days from discharge, FH-VTE was associated with VTE (HR, 1.49; 95% CI, 1.24-1.79). An FH-VTE reduced major bleeding risk during the first 7 days after discharge (HR, 0.78; 95% CI, 0.66-0.91) but not thereafter (HR, 1.10; 95% CI, 0.84-1.44). Postoperative VTE heritability (SE) was 20% (6%).

**Conclusions and Relevance:**

Familial and most likely genetic factors appear to affect VTE and major bleeding risk following hip and knee replacement surgical procedures. Prolonged VTE prophylaxis might be beneficial in predisposed individuals. There may be a possible evolutionary advantage of prothrombotic genes protecting against traumatic bleeding.

## Key Points

**Question:**

Is a family history of venous thromboembolism associated with postoperative venous thromboembolism and major bleeding in patients with primary hip or knee replacement surgical procedures?

**Findings:**

In this cohort study of 69 505 Swedish patients, a family history of venous thromboembolism was associated with statistically significant increased venous thromboembolism risk and reduced bleeding risk after hip and knee replacement surgical procedures. The heritability (SE) for postoperative venous thromboembolism was 20% (6%).

**Meaning:**

Familial and most likely genetic factors appear to affect venous thromboembolism and major bleeding risk following hip and knee replacement surgical procedures.

## Teaser

This cohort study uses Swedish national databases to assess major bleeding and venous thromboembolism (VTE) following hip and knee replacement surgical procedures in patients with and without a family history of VTE.

## Introduction

Hip and knee replacement operations are generally safe procedures but do carry a small risk of serious complications.^1^ The risk of venous thromboembolism (VTE) and major bleeding with pharmacologic thromboprophylaxis has been studied extensively.^2^ VTE risk has been estimated to be 1.15% within 90 days of postoperative care in contemporary trials since 2003.^2^ After 90 days, the VTE risk returns to the baseline risk before surgery.^2^ Major bleeding rates have been reported to be between 0.1% and 3.1% in prevention trials of patients undergoing hip arthroplasty and between 0.2% to 1.4% for patients undergoing knee arthroplasty.^3^ A large population-based study found a total VTE risk of 1.3% and a major bleeding risk of 0.6%.^4^ Although studies have examined predictors associated with VTE, data on predictors associated with major bleeding are sparse.^4,5,6^ The involvement of genetic factors in postoperative thrombosis is not clear. A study by Svensson et al^7^ found an association between factor V Leiden (FVL), single-nucleotide polymorphism rs6025, and VTE risk among patients undergoing hip arthroplasty with short-term low-molecular-weight–heparin (LMWH) prophylaxis during hospitalization but not in those patients with prolonged LMWH prophylaxis (exactly 3 weeks). A study by Wåhlander et al^8^ of patients with a hip or knee replacement surgical procedure found an increased risk for VTE in patients with the prothrombin gene G20210A variant (single-nucleotide polymorphism rs1799963) but not in patients with FVL. However, a study by Ryan et al^9^ found no association between FVL and VTE in patients after joint replacement surgical procedures. Thus, the results of genetic studies for postoperative VTE are divergent.

Family history of VTE (FH-VTE) is a risk factor in first-degree relatives and is associated with 2 to 3 times the increased familial relative risk.^10,11^ However, to our knowledge, FH-VTE has only been investigated in joint replacement surgical procedures in 1 small study (13 VTE cases).^12^ Familial aggregation represents the sum of shared family environmental and genetic factors.^10,11^ However, previous studies indicate a weak involvement of shared environmental factors to the familial aggregation of VTE.^10,11^ The FH-VTE is therefore an important research tool and an important risk factor for VTE reflecting a potential genetic predisposition.^10,11^

In this nationwide Swedish cohort study, we examined the risk of VTE and major bleeding associated with an FH-VTE within 90 days following primary hip and knee replacement surgical procedures.

## Methods

We linked Swedish registers and health care data from several sources to form a data set.^13,14,15,16,17,18,19^ This linkage was based on the Swedish personal identity number. The identity numbers were replaced with serial numbers to preserve confidentiality. Our data set contained the following data sources: the Total Population Register; the Swedish Prescribed Drug Register; the Swedish Inpatient Register (IPR), which included all hospitalizations in Sweden between 1964 and 2012; the Swedish Multi-Generation Register; the Outpatient Register (OPR), which included information from all hospital outpatient visits in Sweden from 2001 through 2012; and the Cause of Death Register. The data analysis was performed from September 1, 2017, through June 15, 2018.

The study was approved by the ethics committee of Lund University, Lund, Sweden. Informed consent was waived as a requirement by the ethics committee of Lund University. This study followed the Strengthening the Reporting of Observational Studies in Epidemiology (STROBE) reporting guideline.

### Sample Population

From the IPR, we included 102 314 Swedish-born patients from the Swedish Multi-Generation Register (ie, born after 1931) who underwent hip or knee replacement surgical procedures between July 1, 2005, and August 31, 2012, identified by surgery procedures coded NFB09, NFB19, NFB29, NFB39, NFB49, NFB62, NFB99, NGB09, NGB19, NGB29, NGB39, NGB49, NGB53, NGB59, and NGB99, according to the Swedish version of the *Classification of Surgical Procedures*.^20^ After the exclusion of patients with previous VTE before the surgical procedure date, a total of 97 226 patients remained. Previous VTE was defined by *International Classification of Diseases* (*ICD*) diagnosis codes of VTE between 1964 and 2012 before the surgical procedure date (eTable 1 in the Supplement). Only individuals with at least 1 biological parent and 1 full sibling alive between 1964 and the surgical procedure index date were included. Thus, 69 505 patients (aged ≥18 years) with hip or knee replacement surgical procedures remained and formed the study cohort.

### VTE and Bleeding

We linked the included patients in the study cohort to the Swedish IPR, OPR, and Cause of Death Register. Medical diagnoses (*ICD-10* diagnosis codes) of pulmonary embolism (I26) and deep venous thrombosis (I80), but not superficial venous thrombosis (I800), were used to identify those 1319 individuals who were diagnosed with VTE within 90 days after the surgical procedure. The validity is high for VTE in the IPR, similar to other diagnoses in the Swedish IPR (85%-95%).^17,18,21,22^ To increase validity, we also required that those patients with a diagnosis of VTE in the OPR and the IPR were prescribed an anticoagulant drug with the following Anatomic Therapeutic Chemical classification system^23^ codes within 30 days from the VTE diagnosis: B01AA01, B01AA03, B01AA04, B01AA07, B01AB01, B01AB04, B01AB05, B01AB09, B01AB10, B01AE05, B01AE07, B01AF01, B01AF02, and B01AX05. We identified 195 of 1319 (14.8%) patients with VTE diagnosis who did not receive anticoagulant drugs, and these patients were not counted as VTE events. After linking to the Multi-Generation Register and excluding those patients without a living parent and full sibling between 1964 and the surgical procedure index date, there were 803 of 69 505 patients (1.2%) diagnosed with VTE within 90 days following the surgical procedure.

Major bleeding was defined by the following *ICD-10* diagnosis codes in the IPR, OPR, and Cause of Death Register within 90 days following the surgical procedure: intracranial bleeding, gastrointestinal bleeding, and other bleeding (eTable 2 in the Supplement).

### Family History of VTE

The FH-VTE predictor variable was defined as the diagnosis of VTE in a parent or full sibling between 1964 and the surgical procedure index date. The IPR, OPR, and Cause of Death Register were used to define VTE in parents and full siblings. The *ICD* diagnosis codes used to define FH-VTE are listed in eTable 1 in the Supplement.

### Adjusting Variables

Patient age at the discharge date was treated as a continuous variable. Augmented Charlson Comorbidity Index (aCCI) was defined by *ICD-10* codes within 4.5 years before the surgical procedure index date (eTable 3 in the Supplement).^24,25^ Patients with aCCI = 0 have no disease, and patients with aCCI = 1 have only 1 disease with the weight = 1. Patients with aCCI = 2 have 2 or more points (ie, between 2 and 37 points). Educational level was dichotomized as fewer than 12 years of education vs 12 or more years of education. Among patients, there were 150 of 69 505 (0.2%) individuals who had not been registered with any form of education, and they were regarded as having the lower educational level.

### Statistical Analysis

Cox proportional hazards regression was used to determine the hazard ratio (HR) of VTE and major bleeding in individuals with FH-VTE compared with those with no family history (NFH) of VTE within 90 days following the surgical procedure. Follow-up days were measured from the discharge date until the date of first registration for the diagnosis of VTE (or major bleeding), death, or the end of follow-up (90 days after discharge), whichever came first. Robust SEs were used to adjust the 95% CIs as some patients came from the same families (5.9%). The assumption of proportional hazards was determined by introducing an interaction term with time and FH-VTE. The assumption of proportionality for a period of time was violated. Time interacted with FH-VTE regarding VTE and major bleeding risk (*P* < .001). We considered time-dependent variables for VTE and major bleeding and used an extended Cox proportional hazards regression model (Heaviside functions).^26^ By using this function, the HR formula yields constant HRs for different time intervals.^26^

*Heritability* is defined as a ratio of variances (ie, the proportion of total variance because of variation in additive genetic factors).^27^ The heritability of a binary trait could be estimated using Falconer regression by presuming a liability threshold model of the disease (ie, whereby everyone has a liability to develop the disease, but only individuals above a threshold value do so).^27,28,29^ To evaluate heritability for postoperative VTE, Falconer regression was used.^28,29^ Using the prevalence rate of the relatives of the first-degree probands (ie, first-degree relatives to affected patients) and the controls (ie, first-degree relatives to unaffected patients) from the case-control study, the heritability (SE) was calculated with the assumption that the contribution of shared environment factors is negligible.^10,11,22,29^

To estimate heritability based on the Falconer method, we used a case-control exact matching method (1:5) by drawing a sample of affected patients as cases with matched control groups of unaffected patients.^30^ The control groups were matched based on sex, birth year, number of full siblings in each family, and educational level. In the case-control study, both groups were linked to their first-degree relatives (parents and full siblings) that were used to calculate postoperative heritability.^29^ Falconer describes the calculation of heritability in detail.^29^ Conditional logistic regression was used for the case-control study. We used SAS, version 9.3 (SAS Institute Inc) and R, version 3.3.2 (R Core Team) for calculating heritability. A 2-sided *P* < .05 was considered statistically significant.

## Results

From the OPR and IPR, we identified 69 505 participants born after 1931 who met the study criterion that they had undergone primary hip or knee replacement surgical procedures from July 1, 2005, through August 31, 2012 (Table 1). Of these participants, 37 989 (54.7%) were women, and the median (interquartile range [IQR]) age for all patients at the date of discharge was 65 (59-70) years (eFigure 1 in the Supplement). A total of 803 of 69 505 patients (1.2%) experienced postoperative VTE and 1285 (1.8%) experienced major bleeding. There was a slight but significant difference in median (IQR) age at the time of the surgical procedure between the NFH group (n = 53 647) and FH-VTE group (n = 15 858) (median [IQR], 65 [59-70] years in NFH group vs 65 [60-70] years in FH-VTE group; *P* < .001). Compared with NFH patients, FH-VTE patients had significantly lower educational levels (3436 of 15 858 [21.7%] FH-VTE patients vs 12 995 of 53 647 [24.2%] NFH patients; *P* < .001); were affected more often with postoperative VTE within 90 days after the surgical procedure (231 [1.5%] vs 572 [1.1%]; *P* < .001); had lower aCCI (10 789 [68.0%] vs 37 327 [69.6%]; *P* = .001); and were less often affected by bleeding within 90 days postoperatively (261 [1.6%] vs 1024 [1.9%]; *P* = .03). The total aCCI distribution in the FH-VTE and NFH groups are shown in eTable 4 in the Supplement. The number of female patients was not significantly different (8597 of 15 858 [54.2%] FH-VTE patients vs 29 392 of 53 647 [54.8%] NFH patients; *P* = .20), and the mean (SD) hospital admission days were also similar (mean [SD], 5.2 [2.7] days vs 5.3 [2.8] days; *P* = .27).

**Table 1.** Characteristics of 69 505 Swedish Patients Who Underwent Hip and Knee Replacement Surgical Procedures Between June 1, 2005, and August 31, 2012

Characteristic	No. (%)			*P* Value
	All (N = 69 505)	NFH (n = 53 647)	FH-VTE (n = 15 858)	
Female	37 989 (54.7)	29 392 (54.8)	8597 (54.2)	.20^a^
Augmented Charlson Comorbidity Index				
0	48 116 (69.2)	37 327 (69.6)	10 789 (68.0)	.001^a^
1	10 953 (15.8)	8350 (15.6)	2603 (16.4)	
2	10 436 (15.0)	7970 (14.9)	2466 (15.6)	
Educational level, ≥12 y	16 431 (23.6)	12 995 (24.2)	3436 (21.7)	<.001^a^
VTE	803 (1.2)	572 (1.1)	231 (1.5)	<.001^a^
Death	343 (0.5)	263 (0.5)	80 (0.5)	.82^a^
Bleeding				
Overall	1285 (1.8)	1024 (1.9)	261 (1.6)	.03^a^
Intracranial	53 (0.1)	39 (0.1)	14 (0.1)	.53^a^
Gastrointestinal	158 (0.2)	123 (0.2)	35 (0.2)	.84
Other	1102 (1.6)	888 (1.7)	214 (1.4)	.01
Age at discharge date, median (IQR), y	65 (59-70)	65 (59-70)	65 (60-70)	<.001^b^
Family size, median (IQR), No.	5 (4-6)	5 (4-6)	5 (4-6)	<.001^b^
Hospitalization time, mean (SD), d	5.3 (2.8)	5.3 (2.8)	5.2 (2.7)	.27^c^

Abbreviations: FH-VTE, family history of venous thromboembolism; IQR, interquartile range; NFH, no family history of venous thromboembolism; VTE, venous thromboembolism.

^a^ χ^2^ test. *P* < .05 is considered significant.

^b^ Kruskal-Wallis (Wilcoxon rank sum score) test.

^c^ Two-sided *t* test. *P* < .05 is considered significant.

### FH-VTE and Postoperative VTE

During a follow-up of 90 days after discharge, 572 of 53 647 (1.1%) NFH patients were diagnosed with VTE (Table 1). The sum of follow-up time for NFH patients was 13 080.4 years, which corresponded to a VTE incidence rate of 43.7 (95% CI, 40.3-47.5) per 1000 person-years. Among FH-VTE patients, 231 of 15 858 (1.5%) were diagnosed with VTE. The sum of follow-up time for FH-VTE patients was 3855.6 years, which corresponded to a VTE incidence rate of 59.9 (95% CI, 52.7-68.2) per 1000 person-years.

The cumulative incidences of VTE for patients with NFH and with FH-VTE are shown in eTable 5, eTable 6, and eFigure 2 in the Supplement. In total, 263 of the 803 (32.8%) patients with VTE within 90 days after discharge were diagnosed with VTE during the first week after discharge from the hospital. In this first week, there was no significant difference in VTE rate between 197 of 53 647 (0.4%) NFH patients and 66 of 15 858 (0.4%) FH-VTE patients (*P* = .38). From 1 week after discharge until the end of the follow-up period, 540 of 803 (67.2%) patients were diagnosed with VTE. Thus, 375 of 53 398 (0.7%) NFH patients were less often diagnosed with VTE than 165 of 15 773 (1.0%) FH-VTE patients from the second week until 90 days after the surgical procedure (*P* < .001).

The survival curves for both NFH and FH-VTE in the Figure show that, compared with NFH, the probability to be diagnosed with VTE during the study was higher for patients with an FH-VTE. The FH-VTE group was associated with a significant increased HR for VTE (HR, 1.36; 95% CI, 1.17-1.59) (Table 2). There was an interaction between time after discharge and FH-VTE for the diagnosis of VTE (*P* < .001) violating the proportional hazards assumption. The FH-VTE group was not associated with VTE during the first week after discharge (HR, 1.13; 95% CI, 0.86-1.49) (Table 2). However, more than 7 days after discharge, FH-VTE was associated with VTE risk (HR, 1.49; 95% CI, 1.24-1.79). Table 2 also shows that FH-VTE and aCCI were predictors associated with VTE. The FH-VTE group was significantly associated with VTE (HR, 1.36; 95% CI, 1.17-1.59), although aCCI had a significant influence only when aCCI = 2 (HR, 1.24; 95% CI, 1.03-1.48 in the univariate model and HR, 1.21; 95% CI, 1.01-1.45 in the multivariate model). There was no interaction noted between FH-VTE and aCCI.

**Figure.**
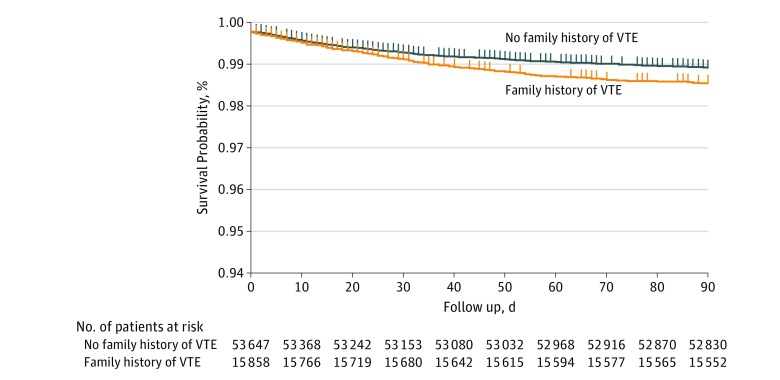
Thrombosis-Free Kaplan-Meier Survival Curves for Patients With and Without a Family History of Venous Thromboembolism (VTE) The probability to be diagnosed with VTE during the study period was higher for patients with a family history of VTE than no family history of VTE (*P* < .001).

**Table 2.** Hazard Ratio for VTE in Patients With a Family History of VTE

Characteristic	Reference	HR (95% CI)	
		Crude	Adjusted^**a**^
Family history	No	1.37 (1.18-1.59)	1.36 (1.17-1.59)
Augmented Charlson Comorbidity Index			
1	0	0.95 (0.78-1.16)	0.94 (0.77-1.15)
2	0	1.24 (1.03-1.48)	1.21 (1.01-1.45)
Age		1.01 (1.00-1.02)	1.01 (1.00-1.01)
Sex	Male	0.94 (0.82-1.08)	0.94 (0.82-1.08)
Educational level	Low	0.98 (0.89-1.07)	0.99 (0.90-1.09)
Family size		1.00 (0.95-1.05)	0.99 (0.95-1.04)
Family history^b^			
Time-divided for VTE ≤7 d after discharge	No	1.13 (0.86-1.49)	1.13 (0.86-1.49)
Time-divided for VTE >7 d until 90 d after discharge	No	1.49 (1.24-1.79)	1.49 (1.24-1.79)

Abbreviations: HR, hazard ratio; VTE, venous thromboembolism.

^a^ Adjusted for augmented Charlson Comorbidity Index, age, sex, educational level, and family size. Low educational level is fewer than 12 years.

^b^ Time-divided HR (95% CI) using extended Cox proportional hazards regression model (Heaviside functions).^26^

### Family History of VTE and Major Bleeding

During a follow-up of 90 days from discharge, 1024 of 53 647 (1.9%) NFH patients were affected by major bleeding. The sum of follow-up time for NFH patients was 12 957.0 years, which corresponds to a major bleeding incidence rate of 79.0 (95% CI, 74.3-84.0) per 1000 person-years. A total of 261 of 15 858 (1.6%) FH-VTE patients were diagnosed with major bleeding. The sum of follow-up time for FH-VTE patients was 3839.9 years, which corresponds to a major bleeding incidence rate of 68.0 (95% CI, 60.2-76.7) per 1000 person-years.

The cumulative incidence of bleeding for those patients with NFH and with FH-VTE are shown in eTable 7, eTable 8, and eFigure 3 in the Supplement. From discharge until 90 days after the surgical procedure, we identified 1285 patients with major bleeding. About 1000 of 1285 (77.8%) bleeding episodes occurred within 1 week after discharge. Thus, within 1 week after discharge, there was a significant difference in bleeding rate between 810 of 53 647 NFH patients (1.51%; 95% CI, 1.41%-1.62%) and 190 of 15 858 FH-VTE patients (1.20%; 95% CI, 1.04%-1.38%) (*P* = .004). However, after 1 week until the end of the follow-up period, only 285 of 1285 (22.2%) major bleeding cases occurred for both types of patients. The bleeding rate after 1 week from discharge was similar for 214 of 52 788 NFH patients (0.4%) and 71 of 15 649 FH-VTE patients (0.5%) (*P* = .40).

The survival curves (not shown) for both NFH and FH-VTE indicated that, compared with NFH, the probability to be diagnosed with major bleeding during the study was higher for the NFH group (*P* = .03). There was an interaction between time and FH-VTE for major bleeding (*P* < .001) violating the proportional hazards assumption. Although FH-VTE was associated with an overall reduced risk of bleeding (HR, 0.84; 95% CI, 0.74-0.97), FH-VTE was associated with reduced bleeding risk only within the first week after discharge (HR, 0.78; 95% CI, 0.66-0.91) but not thereafter (HR, 1.10; 95% CI, 0.84-1.44) (Table 3).

**Table 3.** Hazard Ratio for Major Bleeding in Patients With a Family History of VTE

Characteristic	Reference	HR (95% CI)	
		Crude	Adjusted^**a**^
Family history	No	0.86 (0.75-0.99)	0.84 (0.74-0.97)
Augmented Charlson Comorbidity Index			
1		1.72 (1.50-1.99)	1.66 (1.44-1.91)
2		2.42 (2.13-2.75)	2.30 (2.02-2.62)
Age		1.03 (1.02-1.04)	1.02 (1.01-1.03)
Sex	Male	1.72 (1.53-1.93)	1.71 (1.52-1.92)
Educational level	Low	0.99 (0.92-1.07)	1.03 (0.96-1.11)
Family size		0.98 (0.95-1.02)	0.99 (0.95-1.03)
Family history^b^			
Time-divided for major bleeding ≤7 d after discharge	No	0.79 (0.68-0.93)	0.78 (0.66-0.91)
Time-divided for major bleeding >7 d until 90 d after discharge	No	1.12 (0.86-1.46)	1.10 (0.84-1.44)

Abbreviations: HR, hazard ratio; VTE, venous thromboembolism.

^a^ Adjusted for augmented Charlson Comorbidity Index, age, sex, educational level, and family size. Low educational level is fewer than 12 years.

^b^ Time-divided HR (95% CI) using extended Cox proportional hazards regression model (Heaviside functions).^26^

### Heritability

A matched (1:5) case-control study was performed to determine the heritability for postoperative VTE (782 cases and 3910 controls) and major bleeding (1245 cases and 6225 controls). Postoperative VTE was associated with VTE in the first-degree relatives (parent and/or full sibling), with an odds ratio of 1.34 (95% CI, 1.12 to 1.59) in patients with at least 1 affected first-degree relative. Postoperative bleeding was significantly associated with at least 1 affected first-degree relative, with an odds ratio of 0.83 (95% CI, 0.71-0.97). By using the Falconer method, heritability was estimated based on VTE in the first-degree relatives. The heritability (SE) for VTE calculated from the case-control study was 20% (6%).

## Discussion

The present study linked FH-VTE to an increased incidence of VTE and decreased risk of major bleeding following the surgical procedures. Hip or knee replacement surgical procedures increased the risk for VTE, but LWMH treatment postoperatively for 7 to 10 days had considerably reduced the postoperative VTE risk. Previously, there have been contradictory data about whether genetic risk factors for VTE (ie, rs6025, rs1799963, and ABO blood type variants) are also risk factors for postoperative VTE.^7,8,9^ The present study suggested that familial and probably genetic factors including common risk variants (rs6025 and rs1799963) were risk factors for postoperative VTE. The present study explained why a previous study did not find an association with FVL and venography-diagnosed VTE after 1 week.^9^ The findings were that FH-VTE, and presumably genetic factors, affected the risk of VTE to a larger degree only after more than 7 days after discharge. This conclusion was similar to a study by Svensson et al^7^ who found that FVL was associated with VTE risk only in patients treated for 1 week with LMWH and not in those patients treated for 3 weeks with LMWH. In Sweden, LMWH prophylactic treatment was provided for 7 to 10 days and not extended (≥3 weeks). FVL (rs6025) and the prothrombin variant (rs1799963) are the most common known strong genetic risk factors for VTE in Sweden that are linked to FH-VTE. Especially FVL might have contributed to our findings because of its high frequency in Sweden.^7,10,11^ The importance of ABO blood type is less well studied in Sweden, but non-O blood type is associated with an approximately 2-fold increased risk of VTE.^11^ It possibly could have been worthwhile to screen for the rs6025, rs1799963, and ABO risk variants and to give those patients who were carriers extended LMWH prophylaxis treatment for 3 weeks. Missense PROS1 variants translating into low protein S levels and/or low protein S activity were more common in the general Swedish population than previously anticipated and might have contributed to the situation.^31^ However, it was also possible to extend prophylaxis in all patients with FH-VTE without genetic analysis because known variants only explain 30% of FH-VTE.^32^

Another finding in the present study was that FH-VTE protected against bleeding. Most bleeding events occurred prior to discharge; FH-VTE was not protective against bleeding after 1 week from discharge (ie, not after LMWH treatment was stopped). The present study was the first study, to our knowledge, to show an evolutionary advantage with FH-VTE. Previously, FVL had been shown to protect against intrapartum and menstrual blood loss.^33,34^ Our findings suggest that the genes collectively associated with FH-VTE conferred a procoagulant and evolutionary advantage in situations with trauma to the body. Speculatively, an evolutionary advantage of FH-VTE with less bleeding may have occurred after an attack from a wild animal or a fellow human or after accidental trauma, with a higher chance to survive and achieve reproduction.

A major part of the association of FH-VTE was because of genetic and not shared familial environmental factors.^10,11^ Thus, genes were important even in thrombogenic situations, such as joint replacement surgical procedures, with a heritability of 20%. This result was lower than estimated in young individuals and in selected families with inherited VTE.^10,11,22^ It is therefore possible that the heritability for postoperative VTE was higher in younger individuals and in other less thrombogenic types of operations than joint replacement surgical procedures.

### Strengths and Limitations

The strength of our study was the large study size using validated nationwide registers provided by the Swedish government bodies of Statistics Sweden and the National Board of Health and Welfare.^13,14,15,16,17,18,19,21,22^ Using the Multi-Generation Registers eliminated the risk for recall bias regarding the definition of FH-VTE. The completeness of Swedish registers was another strength. A further strength was the validation of all VTE events with a prescription of anticoagulant treatment. The study was performed in Sweden on individuals born in Sweden; whether the results may be generalized to other populations remains to be determined. However, the diagnostic criteria for VTE used in Sweden was the same as in other countries, and Swedish people are closely related to other western populations.^18^

A limitation was the lack of biological data such as obesity, patient height, and smoking status. However, we adjusted for educational level that was related to lifestyle factors.^35^ As in all epidemiological studies, there still might be residual confounders. Another limitation was that we had no information on the exact treatment time for postoperative LMWH prophylaxis. It is possible that individuals with an FH-VTE were more prone to be treated for a longer time with LMWH because of family awareness. This factor could have diluted our findings with underestimations of the postoperative VTE but also underestimation of the protective effect of FH-VTE against major bleeding. However, most patients undergoing hip and knee replacement surgical procedures in Sweden were treated with LMWH postoperatively for 7 to 10 days. Just as the patients with VTE before the surgical procedures were excluded, several patients with thrombosis at a young age were not included, which may have underestimated the importance of genetic factors. The high prevalence of FH-VTE (22.8%) might also be related to the inclusion of individuals with at least 1 parent and 1 full sibling alive after 1964. The inclusion of superficial thrombophlebitis in the definition of FH-VTE further increased the prevalence of FH-VTE.

Another potential limitation was use of the aCCI, which may not always adequately adjust for all risk factors for VTE and major bleeding. However, the aCCI allowed adjustment for the sum of a large number of comorbidities that would not have been possible otherwise. There was a tendency for fewer comorbidities and lower educational attainments among those with FH-VTE (Table 1). The reason for this occurrence was unclear but might have been related to the clustering of low educational level and comorbidities in families with FH-VTE. Moreover, *ICD* codes for bleeding had not been validated. However, the general validity for the Swedish National Patient register was approximately 85% to 95%.^17^

Another limitation concerned familial risk and heritability. Family history of a disease may have occurred because of genes or the environment, lifestyle, or social factors shared by relatives. Thus, applying Falconer regression had limitations. Structural equation modeling using twins, adoptees, and families was more powerful. However, we knew from previous studies that genetic factors made a strong contribution to the familial transmission of VTE.^10,11,22,36,37,38,39^ In the present study, family history was used as a proxy for genetic defects, but the question remained as to which defects were responsible for the outcomes. For instance, FVL was common in Sweden.^40^

## Conclusions

Family history of VTE appears to have predictive associations with postoperative VTE, and FH-VTE might also have provided protection against major bleeding after joint replacement surgical procedures. This study suggests that extended LMWH treatment might be beneficial in genetically predisposed individuals. We also hypothesize a possible evolutionary advantage of prothrombotic genes protecting against traumatic bleeding.

## Notes

Supplement. **eTable 1.** *ICD (International Classification of Diseases)* Codes Used to Define a Previous History of VTE (Venous Thromboembolism) and Also for Family History of VTE **eTable 2.** *ICD-10 (International Classification of Disease)* Codes Used to Define Major Bleedings **eTable 3.** *ICD-10* Codes for Diagnosis and Weighted Index of Comorbidity Augmented Charlson Comorbidity Index (aCCI) **eTable 4.** Distribution of Total Augmented Charlson Comorbidity Index (aCCI) Among Those With (FH-VTE) and Without (NFH) Family History of Venous Thromboembolism (VTE) **eTable 5.** Cumulative Incidence Probability (CIP) of Venous Thromboembolism (VTE) and 95% Confidence Interval (CI) Among Patients Without Family History of VTE (NFH) (n = 53 647) **eTable 6.** Cumulative Incidence Probability (CIP) of Venous Thromboembolism (VTE) and 95% Confidence Interval (CI) Among Patients With Family History of VTE (FH-VTE) (n = 15 858) **eTable 7.** Cumulative Incidence Probability (CIP) of Bleeding and 95% Confidence Interval (CI) Among Patients Without Family History of VTE (NFH) (n = 53 647) **eTable 8.** Cumulative Incidence Probability (CIP) of Bleeding and 95% Confidence Interval (CI) Among Patients With Family History of VTE (FH-VTE) (n = 15 858) **eFigure 1.** Age at Discharge for All Patients Who Had a Primary Hip and Knee Replacement Surgery Procedure **eFigure 2.** Cumulative Incidence of Venous Thromboembolism (VTE) for Those With (FH-VTE, Red Curve) and Without (NFH, Blue Curve) Family History of Venous Thromboembolism **eFigure 3.** Cumulative Incidence of Bleeding for Those With (FH-VTE, Red Curve) and Without (NFH, Blue Curve) Family History of Venous Thromboembolism
