# Supplementary material for: Postoperative Joint Replacement Complications in Swedish Patients With a Family History of Venous Thromboembolism
Source: JAMA Netw Open. 2018 Sep 7;1(5):e181924. doi: 10.1001/jamanetworkopen.2018.1924 (PMC6324480; doi:10.1001/jamanetworkopen.2018.1924)
Supplement: Supplement. — eTable 1. ICD (International Classification of Diseases) Codes Used to Define a Previous History of VTE (Venous Thromboembolism) and Also for Family History of VTE eTable 2. ICD-10 (International Classification of Disease) Codes Used to Define Major Bleedings eTable 3. ICD-10 Codes for Diagnosis and Weighted Index of Comorbidity Augmented Charlson Comorbidity Index (aCCI) eTable 4. Distribution of Total Augmented Charlson Comorbidity Index (aCCI) Among Those With (FH-VTE) and Without (NFH) Family History of Venous Thromboembolism (VTE) eTable 5. Cumulative Incidence Probability (CIP) of Venous Thromboembolism (VTE) and 95% Confidence Interval (CI) Among Patients Without Family History of VTE (NFH) (n = 53 647) eTable 6. Cumulative Incidence Probability (CIP) of Venous Thromboembolism (VTE) and 95% Confidence Interval (CI) Among Patients With Family History of VTE (FH-VTE) (n = 15 858) eTable 7. Cumulative Incidence Probability (CIP) of Bleeding and 95% Confidence Interval (CI) Among Patients Without Family History of VTE (NFH) (n = 53 647) eTable 8. Cumulative Incidence Probability (CIP) of Bleeding and 95% Confidence Interval (CI) Among Patients With Family History of VTE (FH-VTE) (n = 15 858) eFigure 1. Age at Discharge for All Patients Who Had a Primary Hip and Knee Replacement Surgery Procedure eFigure 2. Cumulative Incidence of Venous Thromboembolism (VTE) for Those With (FH-VTE, Red Curve) and Without (NFH, Blue Curve) Family History of Venous Thromboembolism eFigure 3. Cumulative Incidence of Bleeding for Those With (FH-VTE, Red Curve) and Without (NFH, Blue Curve) Family History of Venous Thromboembolism [file jamanetwopen-1-e181924-s001.pdf]

Supplementary Online Content

Zöller B, Svensson PJ, Sundquist J, Sundquist K, Pirouzifard M. Postoperative joint replacement complications in Swedish patients with a family history of venous thromboembolism. *JAMA Netw Open*. 2018;1(5):e181924. doi:10.1001/jamanetworkopen.2018.1924

eTable 1. *ICD (International Classification of Disease)* Codes Used to Define Previous History of VTE (Venous Thromboembolism) and Also for Family History of VTE

eTable 2. *ICD (International Classification of Disease)* Codes Used to Define Major Bleedings

eTable 3. *ICD-10* Codes for Diagnosis and Weighted Index of Comorbidity Augmented Charlson Comorbidity Index (aCCI)

eTable 4. Distribution of Total Augmented Charlson Index (aCCI) Among Those With (FH-VTE) and Without (NFH) Family History of Venous Thromboembolism (VTE)

eTable 5. Cumulative Incidence Probability (CIP) of Venous Thromboembolism (VTE) and 95% Confidence Interval (CI) Among Patients Without Family History of VTE (NFH) (n=53 647)

eTable 6. Cumulative Incidence Probability (CIP) of Venous Thromboembolism (VTE) and 95% Confidence Interval (CI) Among Patients With Family History of VTE (FH-VTE) (n=15 858)

eTable 7. Cumulative Incidence Probability (CIP) of Bleeding and 95% Confidence Interval (CI) Among Patients Without Family History of VTE (NFH) (n=53 647)

eTable 8. Cumulative Incidence Probability (CIP) of Bleeding and 95% Confidence Interval (CI) Among Patients With Family History of VTE (FH-VTE) (n=15 858)

eFigure 1. Age at Discharge for All Patients Who Had a Primary Hip and Knee Replacement Surgery Procedure

eFigure 2. Cumulative Incidence of Venous Thromboembolism (VTE) for Those With (FH-VTE, Red Curve) and Without (NFH, Blue Curve) Family History of Venous Thromboembolism

eFigure 3. Cumulative Incidence of Bleeding for Those With (FH-VTE, Red Curve) and Without (NFH, Blue Curve) Family History of Venous Thromboembolism

© 2018 Zöller B et al. *JAMA Network Open*.

**eTable 1.** *ICD (International Classification of Disease)* Codes Used to Define Previous History of VTE (Venous Thromboembolism) and Also For Family History of VTE			
**ICD-10**	**ICD-9**	**ICD-8**	**ICD-7**
I80, I81, I82, I26, I636, I676, O222, O223, O225, O229, O870, O871, O873, O879, O082, O087, O882	451, 452, 453, 437G, 415B, 416W, 671C, 671D, 671E, 671F, 671X, 639G, 673C	321, 450, 451, 452, 453, 671, 673.9	463, 464, 465, 466, 682, 684, 583.00, 334.40, 334.50

© 2018 Zöller B et al. *JAMA Network Open*.

**eTable 2.** *ICD-10 (International Classification of Disease*) Codes Used to Define Major Bleedings		
Intracranial bleeding	Gastrointestinal (GI) bleeding	Other bleedings
I60-I62, S064-S066	I983, K250, K252, K254, K256, K260, K262, K264, K266, K270, K272, K274, K276, K280, K282, K284, K286, K625, K920-922	D500, D508-509, D62, H365, H922, N02, N938-939, R04, R310

© 2018 Zöller B et al. *JAMA Network Open*.

eTable 3. *ICD-10* Codes for Diagnosis and Weighted Index of Comorbidity Augmented Charlson Comorbidity Index (aCCI).

Assigned weights for each condition that a patient has. The total equals the score. Patients with aCCI= 0 have no disease, patients with aCCI =1 have only one disease with the weight=1. Patients with aCCI=2 have two or more points, i.e. between 2 and 37 points

Assigned weights for disease	Conditions	ICD 10 codes
**1**	**Myocardial infarction**	I21; I22; I23
**1**	**Congestive heart failure**	I50; I11.0; I13.0; I13.2
**1**	**Peripheral vascular disease**	I70; I71; I72; I73; I74; I77
**1**	**Cerebrovascular disease**	I60-I69; G45; G46
**1**	**Dementia**	F00-F03; F05.1; G30
**1**	**Chronic pulmonary disease**	J40-J47; J60-J67; J68.4; J70.1; J70.3; J84.1; J92.0; J96.1; J98.2; J98.3
**1**	**Connective tissue disease**	M05; M06; M08; M09; M30; M31; M32; M33; M34; M35; M36; D86
**1**	**Ulcer disease**	K22.1; K25-K28
**1**	**Mild liver disease**	B18; K70.0-K70.3; K70.9; K71; K73; K74; K76.0
**1**	**Diabetes mellitus**	E10.0; E10.1; E10.9; E11.0; E11.1; E11.9
**2**	**Hemiplegia**	G81; G82
**2**	**Moderate/sever renal disease**	I12; I13; N00-N05; N07;N11; N14; N17-N19; Q61
**2**	**Diabetes mellitus with chronic complications**	E10.2-E10.8; E11.2-E11.8
**2**	**Any tumor**	C00-C75
**2**	**Leukemia**	C91-C95
**2**	**Lymphoma**	C81-C85; C88; C90; C96
**3**	**Moderate / sever liver disease**	B15.0; B16.0; B16.2; B19.0; K70.4; K72; K76.6; I85
**6**	**Metastatic solid tumor**	C76-C80
**6**	**AIDS**	B21-B24

© 2018 Zöller B et al. *JAMA Network Open*.

eTable 4. Distribution of Total Augmented Charlson Index (aCCI) Among Those With (FH-VTE) and Without (NFH) Family History of Venous Thromboembolism (VTE)

**Total aCCI**	NFH n (%)<br>53 647	FH-VTE n (%)<br>15 858
**0**	37 327<br>(69.58)	10 789<br>(68.04)
**1**	8 350<br>(15.56)	2 603<br>(16.41)
**2**	4 584<br>(8.54)	1 421<br>(8.96)
**3**	1 621<br>(3.02)	525<br>(3.31)
**4**	641<br>(1.19)	198<br>(1.25)
**5**	297<br>(0.55)	89<br>(0.56)
**6**	166<br>(0.31)	52<br>(0.33)
**7**	62<br>(0.12)	19<br>(0.12)
**8**	406<br>(0.76)	100<br>(0.63)
**9**	120<br>(0.22)	35<br>(0.22)
**10**	43<br>(0.08)	14<br>(0.09)
**11**	16<br>(0.03)	6<br>(0.04)
**12**	7<br>(0.01)	4<br>(0.03)
**13**	6<br>(0.01)	0<br>(0.00)
**14**	1<br>(0.00)	3<br>(0.02)

© 2018 Zöller B et al. *JAMA Network Open*.

eTable 5. Cumulative Incidence Probability (CIP) of Venous Thromboembolism (VTE) and 95% Confidence Interval (CI) Among Patients Without Family History of VTE (NFH) (n=53 647)

Follow-up time interval (days)	Number of events	Number of censored patients	Number at risk at next time interval	CIP (95% CI)
0 – 7	197	52	53 398	0.37 (0.32 – 0.42)
7 – 30	189	66	53 143	0.72 (0.65 – 0.80)
30 – 60	119	60	52 964	0.94 (0.86 – 1.03)
60 – 80	50	48	52 866	1.04 (0.95 – 1.13)
80 – 90	17	52849	0	1.07 (0.98 – 1.16)

© 2018 Zöller B et al. *JAMA Network Open*.

eTable 6. Cumulative Incidence Probability (CIP) of Venous Thromboembolism (VTE) and 95% Confidence Interval (CI) Among Patients With Family History of VTE (FH-VTE) (n=15 858)

Follow-up time interval (days)	Number of events	Number of censored patients	Number at risk at next time interval	CIP (95% CI)
0 – 7	66	19	15 773	0.42 (0.33 – 0.53)
7 – 30	74	23	15 676	0.88 (0.75 – 1.04)
30 – 60	65	18	15 593	1.30 (1.13 – 1.48)
60 – 80	18	11	15 564	1.41 (1.24 – 1.61)
80 – 90	8	15556	0	1.46 (1.29 – 1.66)

© 2018 Zöller B et al. *JAMA Network Open*.

eTable 7. Cumulative Incidence Probability (CIP) of Bleeding and 95% Confidence Interval (CI) Among Patients Without Family History of VTE (NFH) (n=53 647)

Follow-up time interval (days)	Number of events	Number of censored patients	Number at risk at next time interval	CIP (95% CI)
0 – 7	810	49	52 788	1.51 (1.41 – 1.62)
7 – 30	88	67	52 633	1.67 (1.57 – 1.79)
30 – 60	69	56	52 508	1.80 (1.69 – 1.92)
60 – 80	34	51	52 423	1.87 (1.76 – 1.98)
80 – 90	23	52400	0	1.91 (1.80 – 2.03)

© 2018 Zöller B et al. *JAMA Network Open*.

eTable 8. Cumulative Incidence Probability (CIP) of Bleeding and 95% Confidence Interval (CI) Among Patients With Family History of VTE (FH-VTE) (n=15 858)

Follow-up time interval (days)	Number of events	Number of censored patients	Number at risk at next time interval	CIP (95% CI)
0 – 7	190	19	15 649	1.20 (1.04 – 1.38)
7 – 30	25	24	15 600	1.36 (1.19 – 1.55)
30 – 60	24	19	15 557	1.51 (1.33 – 1.71)
60 – 80	12	9	15 536	1.58 (1.40 – 1.79)
80 – 90	10	15526	0	1.65 (1.46 – 1.86)

© 2018 Zöller B et al. *JAMA Network Open*.

eFigure 1. Age at Discharge for All Patients Who Had a Primary Hip and Knee Replacement Surgery Procedure

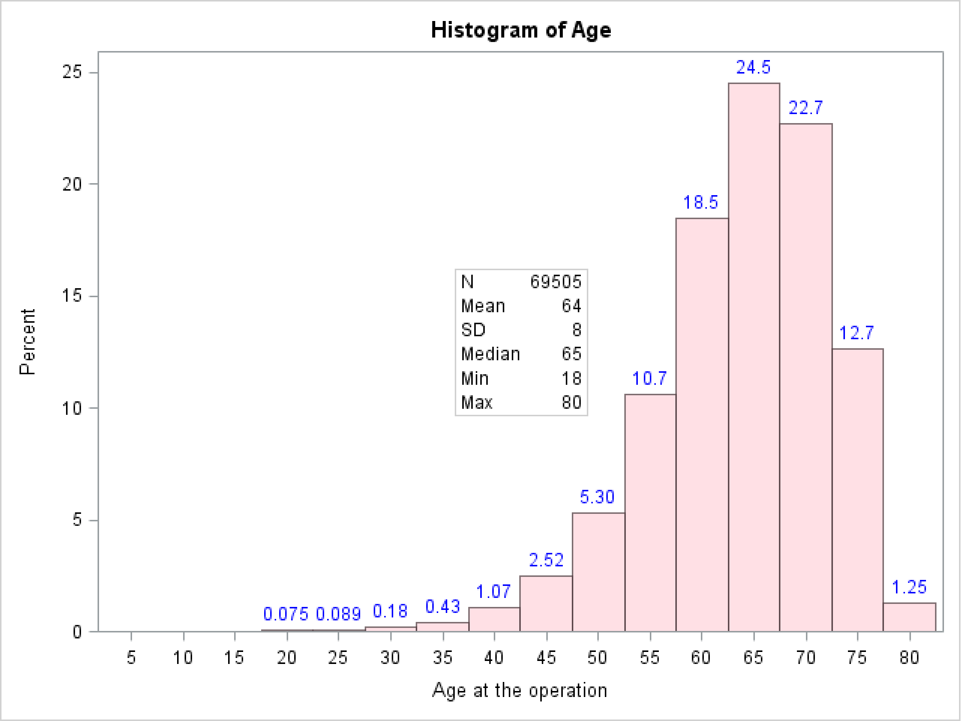

© 2018 Zöller B et al. *JAMA Network Open*.

eFigure 2. Cumulative Incidence of Venous Thromboembolism (VTE) for Those With (FH-VTE, Red Curve) and Without (NFH, Blue Curve) Family History of Venous Thromboembolism

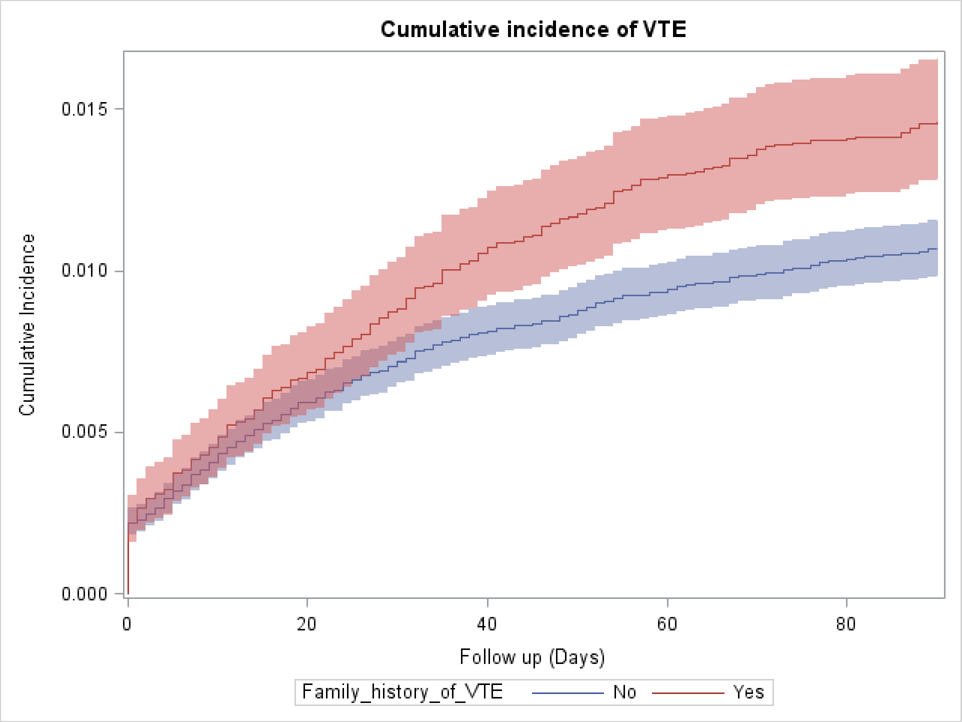

© 2018 Zöller B et al. *JAMA Network Open*.

eFigure 3. Cumulative Incidence of Bleeding for Those With (FH-VTE, Red Curve) and Without (NFH, Blue Curve) Family History of Venous Thromboembolism

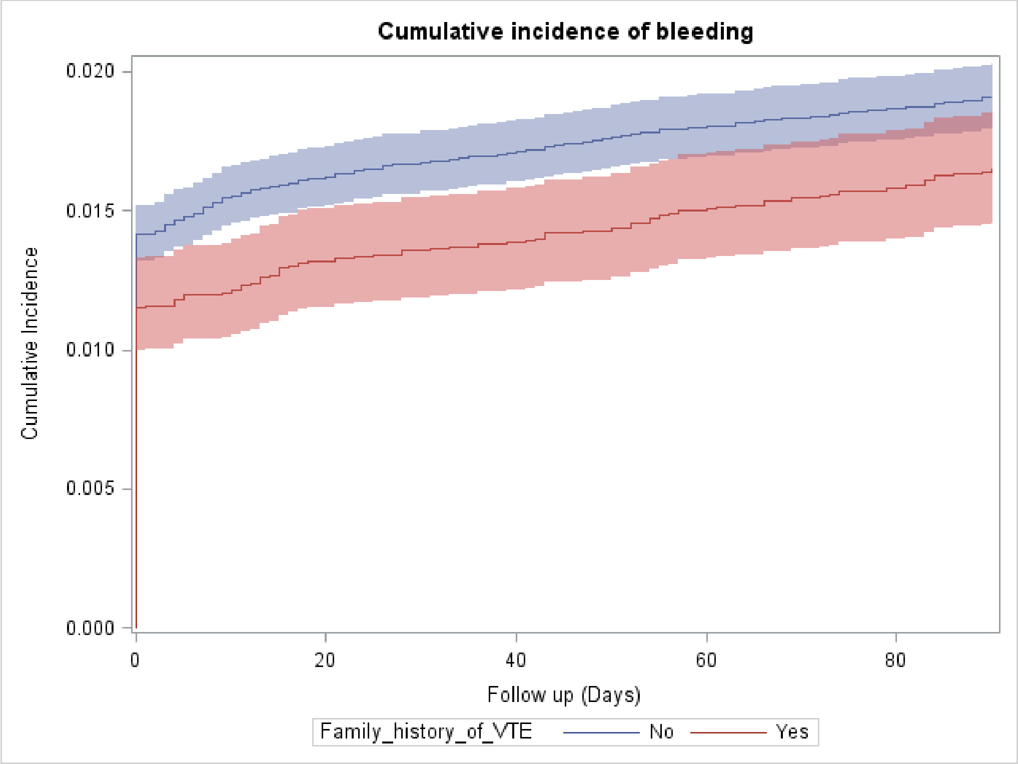

© 2018 Zöller B et al. *JAMA Network Open*.
